# Evaluating the Feasibility of an Electronic Patient-Reported Outcomes Platform Integrating Electronic Health Records and a Mobile Messaging App in Breast Cancer Radiotherapy: Retrospective Cross-Sectional Study

**DOI:** 10.2196/67514

**Published:** 2026-02-05

**Authors:** Jong Yun Baek, Haeyoung Kim, Won Kyung Cho, Nalee Kim, Tae Hoon Lee, Won Chul Cha

**Affiliations:** 1 Department of Radiation Oncology, Samsung Medical Center, Sungkyunkwan University School of Medicine Seoul Republic of Korea; 2 Department of Digital Health, Samsung Advanced Institute for Health Sciences & Technology, Sungkyunkwan University Seoul Republic of Korea; 3 Data Innovation Center, Samsung Medical Center, Seoul, Republic of Korea Seoul Republic of Korea

**Keywords:** patient-reported outcomes, mobile app, mHealth, mobile messaging app, electronic health records, eHealth, telemedicine, radiotherapy, breast neoplasms

## Abstract

**Background:**

Integrating electronic patient-reported outcomes (ePROs) into electronic health records (EHRs) can enhance the quality of patient care. However, collecting longitudinal ePRO data throughout treatment and posttreatment surveillance remains challenging in patients with breast cancer. To address this, we implemented an automated system that enables ePRO acquisition and seamless integration into the EHR. The system delivers questionnaire weblinks via a mobile messaging app, allowing patients to complete ePROs before clinic visits, with responses automatically transferred to the EHR.

**Objective:**

This study aimed to assess patient response rates to the ePRO system and identify key factors influencing the response rate among patients with breast cancer who received radiotherapy and postradiotherapy follow-up.

**Methods:**

We conducted a retrospective analysis of prospectively collected ePRO data by using the BREAST-Q questionnaire, a validated patient-reported outcome measure for breast surgery, from patients who received adjuvant radiotherapy at our institution between May 2023 and April 2024. At a preradiotherapy or postradiotherapy visit, each patient was asked to complete the questionnaire via a weblink sent to their mobile messaging app, KakaoTalk. The questionnaire was dispatched from minutes to several days before each visit. The response rate was calculated as the percentage of patients whose responses were successfully recorded in the EHR among those who were requested to respond. A complete response (CR) was defined as completion of all required questionnaire items. CR rates were analyzed according to clinical factors using univariate and multivariate logistic regression.

**Results:**

Data from 1488 patients were analyzed, encompassing 2431 encounters (median 1, IQR 1-2 per patient). The median age of the patients was 51 (range 23-83) years, with 65.1% (n=968) patients aged 40 to 59 years. Comorbidities were present in 15% (223/1488) of the patients. The CR rate for the first, second, and third ePRO encounters was 89.9% (1338/1488), 98.3% (735/748), and 97.3% (180/185), respectively. Among first-time respondents, younger patients had a significantly higher CR rate (patients aged <60 years: 100/1104, 90.9%; patients aged ≥60 years: 334/384, 87%; *P*=.03). The timing of the questionnaire dispatch also affected the CR rate (*P*<.001). The CR rate was the highest when questionnaires were sent more than 1 hour before the visit (547/583, 93.3%) or in the afternoon of the previous day (505/545, 92.7%) and the lowest when sent 2 or more days before (100/130, 76.9%) or within 1 hour before the appointment (92/112, 81.7%). Both age (*P*=.006) and timing (*P*<.001) remained significant in the multivariate analysis.

**Conclusions:**

This study demonstrates the feasibility of integrating ePRO into EHR through a mobile messaging app–based system, with high patient adherence. Response rates were significantly influenced by patient age and the timing of questionnaire dispatch. These findings provide practical insight for optimizing ePRO implementation in routine oncology care.

## Introduction

### Background

Breast radiotherapy is an essential element in the management of breast cancer, as it enables breast conservation by eliminating microscopic tumor foci following tumor resection and prevents locoregional recurrence, leading to improved survival [1,2]. With advances in multimodality treatment, survival outcomes for patients with breast cancer have substantially improved over the past decades [3,4]. As survival extends, radiotherapy regimens have evolved to minimize treatment-related toxicity while maintaining tumor control [5]. Therefore, capturing toxicity profiles and patient satisfaction is critical for optimizing radiotherapeutic approaches. To achieve this, the incorporation of patient-reported outcomes (PROs) has become increasingly important for individualized counseling and shared decision-making [6].

Despite growing consensus on the value of electronic PROs (ePROs), their routine adoption in oncology remains limited. Collecting longitudinal ePRO data across active treatment and long-term follow-up is particularly challenging, as survivors of breast cancer often require monitoring for more than a decade after completion of primary treatment [7,8]. Previous studies have highlighted several barriers to sustained ePRO use, including workflow burden on clinicians, lack of integration with electronic health records (EHRs), and patient fatigue over repeated reporting [9-12]. Most existing ePRO systems operate as stand-alone or web portal–based platforms, requiring separate log-ins or additional applications, which hinders seamless use during clinical visits [9-11]. As a result, adherence to ePRO completion varies widely, ranging from 27% to 95% across populations with cancer [13-17], and evidence on how to maintain high adherence in daily oncology practice is still lacking.

To address these gaps, we developed and implemented an automated EHR-integrated ePRO system that delivers questionnaires via KakaoTalk (Kakao Corp), the most widely used mobile messaging app in South Korea [18]. This study evaluated the feasibility of this platform by assessing response rates and identifying clinical and contextual factors associated with ePRO adherence among patients receiving postoperative adjuvant breast radiotherapy and follow-up. For an assessment of the feasibility of the ePRO system, the response rate was considered the primary indicator, as it reflects both patient adherence and the sustainability of the platform in routine clinical practice. Consistent with previous ePRO implementation studies [9,10,14,15,17], the response rate has been widely used as a practical measure of feasibility, representing patient engagement, system usability, and operational sustainability within clinical workflows. A consistently high response rate across visits would indicate that the system can be feasibly integrated into long-term follow-up, whereas lower or variable rates would highlight barriers that require further optimization. In addition, factors influencing feasibility were examined by analyzing both clinical and contextual variables that might affect adherence. Clinical variables, such as age, type of surgery, histology, and comorbidities, were considered because they may influence treatment-related experiences and patients’ ability to engage with the ePRO systems. Contextual factors, including previous exposure to other ePRO systems and the timing of questionnaire requests, were analyzed because they are expected to directly influence adherence and response behavior.

### Study Objective

This study aimed to evaluate the feasibility of integrating an ePRO system directly into the EHR through a mobile messaging app by assessing patient adherence and identifying clinical and contextual factors associated with ePRO completion among patients with breast cancer undergoing postoperative adjuvant radiotherapy and follow-up.

## Methods

### Participants and Study Design

This study was conducted at the Samsung Medical Center, a large tertiary referral hospital in South Korea, with a comprehensive cancer center located in Seoul. Located in an urban area, the institution serves as a nationwide referral center, providing care to a high volume of patients with cancer from both urban and rural regions. As of 2021, approximately 31,000 patients—representing about 11% of all patients with cancer in South Korea—were treated at the Samsung Medical Center [19]. Between May 2023 and April 2024, ePRO questionnaires were distributed to patients visiting the Department of Radiation Oncology for postoperative adjuvant radiation treatment for breast cancer. These visits included both preradiotherapy evaluations and postradiotherapy follow-ups for surveillance. A weblink connected to the ePRO questionnaires was sent to each individual’s KakaoTalk mobile messaging app before their appointment with the attending physician, and patients were asked to respond to the questionnaires through the app. KakaoTalk is a free mobile messaging app used by more than 90% of the Korean population across all age groups and has been widely incorporated into both personal and institutional services. Its functions extend beyond instant messaging to include secure log-in verification, payment systems, and government or health care notifications [20,21]. Because of its nationwide penetration and user familiarity, the app enables seamless integration of digital health tools, such as ePRO questionnaires, without requiring additional software installation or user registration. The dispatch of the questionnaires was managed by nurses or physicians through a system deployed in the EHR before each patient’s visit. The timing of sending the questionnaire varied depending on the sender’s preference, ranging from days to minutes before the visit, with no predefined time points. On the day of the visit, an outpatient receptionist or nurse checked whether the questionnaires had been completed. If the questionnaires were incomplete, the receptionist or nurse briefly asked the patient to complete them before the physician’s session.

During the study period, the adaptation of the ePRO platform into daily clinical practice varied among the radiation oncologists in our department. Some began using the platform at the beginning of the study period, while others adopted it later. Once a radiation oncologist initiated use of the system, all eligible patients assigned to them were requested to complete the ePRO questionnaires, regardless of individual characteristics. As a result, not all patients with breast cancer visiting our department were uniformly invited to complete ePRO questionnaires. Instead, the number of patients with breast cancer undergoing radiotherapy who were requested to submit ePROs increased toward the latter part of the study period.

After an outpatient visit for preradiotherapy evaluation, patients received radiation treatment according to our institutional protocol. Radiotherapy was administered once daily for 5 consecutive days, with 3 to 19 fractionations over a period of 1 to 4 weeks. Fractionation schedules were determined based on tumor stage, surgery types, the inclusion of regional nodal irradiation, and other risk factors. After completing the treatment, patients were followed up for 2 to 3 weeks after treatment and subsequently every 6 months. ePRO data were collected at the preradiotherapy visit, the immediate postradiotherapy visit at 2 to 3 weeks after treatment, and every 6 months thereafter. As each patient was required to respond to a questionnaire at each hospital visit, 1 or more questionnaires were requested to be completed by each patient during the period of this study.

### A System for ePRO Collection and Storage in the EHR

A system for ePRO acquisition and integration with the EHR was developed as an in-house model at our institution. The system links our hospital’s EHR with the individual’s mobile messaging app for collecting and storing ePRO data. Data entry is performed through the messaging app on the patient’s mobile phone, while the data presentation and storage are conducted in our hospital’s EHR. A section dedicated to ePRO is integrated into the EHR, allowing physicians and other medical staff to send new ePRO questionnaires on request and view each patient’s responses at any time (Figure S1 in Multimedia Appendix 1). When medical staff select ePRO questionnaires and dispatch them through the EHR, a weblink for the ePRO questionnaires is sent to the patient’s messaging app. The patient can open the link by entering their date of birth and submit their response to the questionnaire, which is in the form of checkboxes (Figure S2 in Multimedia Appendix 1). The patient’s responses are automatically transferred to the ePRO section of the EHR and stored in the hospital’s data warehouse. This process of entering, transferring, and storing ePRO data occurs simultaneously in real time, enabling physicians to view the content and time stamp of the data in the EHR.

For the PRO instrument used in this study, we used the Korean version of BREAST-Q (version 2.0) postoperative scale, including modules for breast-conserving therapy, mastectomy, and reconstruction. Among the domains of these modules, the following were used for our patients: satisfaction with breasts, satisfaction with implants, physical well-being of the chest or upper body, and adverse effects of radiation [22,23]. Patients who visited for preradiotherapy evaluation were asked to complete a questionnaire without the domain of adverse effects of radiation, while those attending for postradiotherapy surveillance received a questionnaire that included the domain.

### Assessment of the Response Rate and Influencing Factors

Response rate was calculated as the percentage of patients whose responses were successfully recorded in the EHR among those who were requested to respond to the questionnaires. The response rate for each survey encounter was assessed and compared according to the number of encounters, from the first to the third. Because the current analysis was based on surveys conducted over 1 year, most of our patients encountered the questionnaires 1 to 3 times according to the scheduled follow-up interval. We classified response status into 3 categories: complete response (CR; all questions answered), partial response (PR; at least 1 question answered but not all), and no response (NR). In addition, when analyzing significant factors influencing CR, we divided our patients into 2 groups: complete responders and noncomplete responders, with partial responders and nonresponders merged into the noncomplete responder group.

To determine the significant factors influencing CR, we compared the CR rate according to various factors, focusing only on patients who encountered the survey for the first time. Specifically, to assess the impact of questionnaire request timing before a visit appointment, the timing was categorized into 5 groups: within 1 hour of the appointment time (≤1 hour on the day), more than 1 hour before the appointment on the visit day (>1 hour on the day), in the afternoon of the day before the appointment (PM the day before), in the morning of the day before the appointment (AM the day before), and 2 or more days before the appointment day (≥2 days before). In addition, patients who had previous experiences responding to questionnaires requested from other departments in our hospital were categorized as previous other ePRO (+), while those without the experiences were categorized as previous other ePRO (–). Finally, patients with any of the following diseases were categorized as having comorbidity: diabetes, cardiovascular disease, chronic pulmonary disease, hepatic disease, renal disease, or other cancers.

### Statistical Analysis

For the univariate analysis, Fisher exact test was used to compare the CR according to clinical and contextual variables, including age, type of surgery, histology, comorbidities, previous ePRO experience, and the timing of questionnaire requests. Age was categorized using a 60-year cutoff for univariate analysis and treated as a continuous variable in multivariate models. Patients with missing data for a given variable were excluded from the study. Factors with *P*<.10 in univariate analyses were included in the multivariate logistic regression model to adjust for potential confounding effects. All *P* values were 2-sided, with *P*<.05 considered statistically significant. All analyses were performed using SPSS software (version 27; IBM Corp).

### Ethical Considerations

This study was approved by the institutional review board of the Samsung Medical Center (institutional review board approval number 2024-07-147-001). The analysis was conducted retrospectively using deidentified ePRO data collected during routine clinical care, and the need for informed consent was waived by the institutional review board. All patient information was deidentified before analysis to ensure privacy and confidentiality. No personal identifiers were included in the downloaded dataset. No compensation was provided to patients for participation, as the data were collected as part of standard clinical procedures without additional burden or intervention.

## Results

### Patients’ Characteristics

A total of 2334 patients with breast cancer attended our department during the study period. Of the 2334 patients, 1491 (63.9%) with nonmetastatic breast cancer were invited to complete the ePRO survey. This difference reflects the gradual adoption of the ePRO system among physicians, as the platform was implemented in stages and not all attending radiation oncologists had begun using it at the start of the study period. A total of 3 (0.2%) patients were excluded from the analysis due to missing time records of their questionnaire responses, resulting in 1488 (99.8%) patients being included in this study. Among them, 740 (49.7%) encountered 1 survey, 563 (37.8%) encountered 2 surveys, 175 (11.8%) encountered 3 surveys, and 10 (0.7%) encountered 4 surveys, resulting in 2431 survey encounters.

The characteristics of the 1488 patients are summarized in Table 1. The median age was 51 years, with most of the patients (n=968, 65.1%) aged between 40 and 59 years, and 41 (2.8%) patients were aged 75 years or older. Most patients underwent breast-conserving surgery (n=1186, 79.7%) and had invasive carcinoma (n=1342, 90.2%). Comorbidities were found in 223 (14.9%) patients, including diabetes (n=98, 6.6%); cardiovascular disease (n=67, 4.5%); chronic disease of the liver, lung, or kidney (n=63, 4.2%); and other cancers (n=26, 1.7%).

**Table 1:** Patients’ characteristics (N=1488).

Characteristics		Patients
Age (y), median (range)		51 (24-85)
**Age (y), n (%)**		
	≥20 to <40	136 (9.1)
	≥40 to <60	968 (65.1)
	≥60	384 (25.8)
**Sex, n (%)**		
	Female	1487 (99.9)
	Male	1 (0.1)
**Type of surgery, n (%)**		
	Breast-conserving surgery	1186 (79.7)
	Mastectomy without reconstruction	129 (8.7)
	Mastectomy with reconstruction	173 (11.6)
**Histology, n (%)**		
	Ductal carcinoma in situ	146 (9.8)
	Invasive carcinoma	1342 (90.2)
**Type of visit, n (%)**		
	Preradiotherapy evaluation visit	946 (63.6)
	Postradiotherapy follow-up visit	542 (36.4)
**Comorbidity, n (%)**		
	Yes	223 (15)
	No	1265 (85)
**Experience of other electronic patient-reported outcomes, n (%)**		
	Yes	392 (26.3)
	No	1096 (73.7)
**Timing of the questionnaire requests^a^, n (%)**		
	≤1 h on the day	115 (7.7)
	>1 h on the day	586 (39.4)
	PM the day before	545 (36.6)
	AM the day before	112 (7.5)
	≥2 d before	130 (8.7)

^a^The timing of the questionnaire requests before a visit appointment was categorized as follows: within 1 hour of the appointment time (≤1 hour on the day), more than 1 hour before the appointment on the visit day (>1 hour on the day), in the afternoon of the day before the appointment (PM the day before), in the morning of the day before the appointment (AM the day before), and 2 or more days before the appointment day (≥2 days before).

### Response Rate and Influencing Factors

**Figure 1:**
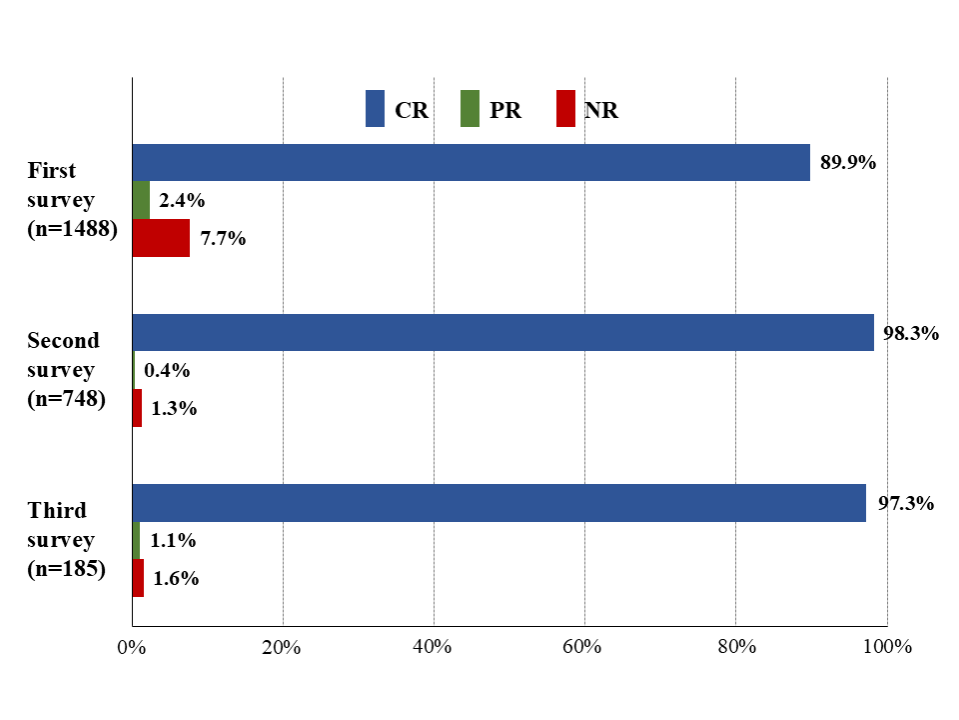
Rates of complete response (CR), partial response (PR), and no response (NR) for electronic patient-reported outcomes surveys across first, second, and third requests.

Of the 1488 patients who encountered the questionnaires for the first time, 1338 (89.9%) exhibited CR, 35 (2.4%) submitted PR, and 115 (7.7%) did not respond to the questionnaire. The response status and rates according to the number of survey encounters are shown in Figure 1.

In the univariate analysis of factors influencing CR, the following were statistically significant: patient’s age, type of visit, and the timing of the questionnaire requests before a visit appointment (Table 2). The CR rate was 90.9% (1004/1104) in patients younger than 60 years and 87% (334/384) in those aged 60 years or older (*P*=.03), indicating that older age was associated with lower completion. A significantly lower CR rate was also observed in patients receiving the questionnaire at the preradiotherapy visit (837/946, 88.5%) compared to the postradiotherapy visits (501/542, 92.4%; *P*=.02).

**Table 2:** Univariate analysis of factors affecting the complete response to the electronic patient-reported outcome questionnaire (N=1488).

Characteristics			CR^a^, n (%)		Non-CR, n (%)		*P* value	
**Age (y)**								.03
	<60	1004 (90.9)		100 (9.1)				
	≥60	334 (87)		50 (13)				
**Type of surgery**								.10
	Breast-conserving surgery	1057 (89.1)		129 (10.9)				
	Mastectomy without reconstruction	122 (94.6)		7 (5.4)				
	Mastectomy with reconstruction	159 (91.9)		14 (8.1)				
**Histology**								.77
	Ductal carcinoma in situ	133 (91.1)		13 (8.9)				
	Invasive carcinoma	1205 (89.8)		137 (10.2)				
**Type of visit**								.02
	Preradiotherapy evaluation visit	837 (88.5)		109 (11.5)				
	Postradiotherapy follow-up visit	501 (92.4)		41 (7.6)				
**Comorbidity**								.55
	Yes	198 (88.8)		25 (11.2)				
	No	1140 (90.1)		125 (9.9)				
**Experience of other electronic patient-reported outcome**								.85
	Yes	354 (90.3)		38 (9.7)				
	No	984 (89.8)		112 (10.2)				
								<.001
	≤1 h on the day	94 (81.7)		21 (18.3)				
	>1 h on the day	547 (93.3)		39 (6.7)				
	PM the day before	505 (92.7)		40 (7.3)				
	AM the day before	92 (82.1)		20 (17.9)				
	≥2 d before	100 (76.9)		30 (23.1)				

^a^CR: complete response.

^b^The timing of the questionnaire requests before a visit appointment was categorized as follows: within 1 hour of the appointment time (≤1 hour on the day), more than 1 hour before the appointment on the visit day (>1 hour on the day), in the afternoon of the day before the appointment (PM the day before), in the morning of the day before the appointment (AM the day before), and 2 or more days before the appointment day (≥2 days before).

Notably, the timing of the questionnaire requests had a strong influence on CR. Patients who received the questionnaire more than 1 hour before the appointment or in the afternoon of the previous day showed the highest CR rates at 93.3% (547/583) and 92.7% (505/545), respectively. In contrast, the lowest rates were seen in those who received the questionnaire 2 or more days before (100/130, 76.9%) and within 1 hour of the appointment (94/115, 81.7%; *P*<.001).

In the multivariate analysis, age and the timing of the questionnaire requests remained significant influencing factors for CR. Old age (odds ratio 0.98, 95% CI 0.96-0.99; *P*=.006) and questionnaire request timing of ≤1 hour on the day of the visit, AM the day before, or ≥2 days before the appointment (odds ratio 0.32, 95% CI 0.22-0.45; *P*<.001) were significantly associated with a lower CR rate (Table 3).

**Table 3:** Multivariate analysis of factors affecting the complete response to the electronic patient-reported outcome questionnaire.

Characteristics	Odds ratio (95% CI)	*P* value
Age (y): continuous	0.98 (0.96-0.99)	.006
Type of surgery: breast-conserving surgery vs mastectomy or reconstruction	0.64 (0.39-1.05)	.08
Type of visit: preradiotherapy evaluation visit vs postradiotherapy follow-up visit	1.33 (0.90-1.97)	.15
Timing of the questionnaire requests^a^: ≤1 h on the day or AM the day before or ≥2 d before vs >1 h on the day or PM the day before	0.32 (0.22-0.45)	<.001

^a^The timing of ePRO requests before a visit appointment was categorized as follows: within 1 hour of the appointment time (≤1 hour on the day), more than 1 hour before the appointment on the visit day (>1 hour on the day), in the afternoon of the day before the appointment (PM the day before), in the morning of the day before the appointment (AM the day before), and 2 or more days before the appointment day (≥2 days before).

## Discussion

### Principal Findings

Patients using our ePRO system, which links a commercial mobile messaging app with our hospital’s EHR, showed an 89.9% (1338/1488) CR rate for the BREAST-Q questionnaires. Responses of patients who visited our radiation oncology department for breast cancer management were successfully recorded. The response rate to the questionnaires increased as the number of survey encounters increased. Age of 60 years or older was associated with a lower rate of CR; however, 87% (334/384) of participants of this age group provided appropriate responses to the questionnaires delivered through their mobile messaging app, even if it was their first time encountering the questionnaires using the app. Notably, the timing of the questionnaire requests significantly influenced the CR rate, with a higher CR rate of more than 92% observed when the questionnaires were requested more than 1 hour before the scheduled visit or in the afternoon of the day before the appointment. Given these findings, our ePRO system shows potential as a feasible platform for ePRO collection and integration with the hospital’s EHR. In addition, the factors identified as significantly affecting the CR rate of the questionnaires can be used to guide improvements in responses to ePRO questionnaires in daily practice.

### Comparison With Prior Work

The significance of PRO measurement in oncology care has been increasingly emphasized in recent years [24]. In this regard, the European Society for Medical Oncology released a clinical practice guideline concerning the use of PRO in the continuum of cancer care, emphasizing the essential role of symptom monitoring via PRO measurements [25]. ePRO offers several advantages, such as greater patient preference and acceptability, lower human resource costs, and higher data quality [9,11]. Various forms of ePRO collection platforms, including web-based and app-based systems, have been developed and used [13,14,26]. In addition to the data collection system, integrating the data into the EHR is essential to facilitate the incorporation of ePRO into clinical practice [9-12].

In this analysis, we found favorable patient adherence to our ePRO system, with more than 89.9% (1338/1488) CR rate for the ePRO questionnaires, even at the first encounter. This promising result is attributed to adopting a system that uses the KakaoTalk messaging app, which was familiar to our patients [18]. Patients were able to access the questionnaires using the existing app on their smartphone, without the need to install an additional app for ePRO. Because the message with the questionnaire link was sent under the hospital’s name, our patients likely accepted it confidently, without concerns about cybercrimes. Moreover, given that more than 94% of Koreans own a smartphone, ePRO questionnaires delivered via the mobile app could effectively encourage responses from our patient population [27]. According to previous studies, ePRO adherence rate among patients with cancer has been reported to range between 27% and 95% [13-17]. In a study conducted in the United States, ePRO adherence rates ranged from 27% to 70%, following administration either on-site via tablet or remotely via a patient portal. There were significant differences in response rates depending on patient age and race, with older patients aged 65 years and older and non-White individuals being negatively associated with adherence to ePRO [14]. Meanwhile, a Japanese study reported a 95% ePRO adherence rate via a mobile messaging app, LINE, from 40 participants, which was similar to our patients’ adherence rate [16]. Considering that LINE is used by more than 70% of the Japanese population, the familiarity with the ePRO acquisition tool likely contributed to the high ePRO adherence in their study [28]. Taken together, these findings suggest that selecting appropriate tools for ePRO administration based on respondents’ demographic or cultural characteristics is essential for achieving favorable ePRO acceptance.

Older age has been reported to be significantly associated with lower adherence to ePRO [13,14,17]. In a prospective study conducted among French patients aged 75 years and older, 26% of the participants accepted ePRO, which was conducted remotely using a web-based app [13]. More than 52% of the participants did not respond to the ePRO due to technological issues, such as a lack of internet access or discomfort with using the internet [13]. In addition, a study performed in the United States showed that patients aged 65 years or older exhibited a 6% decrease in adherence to ePRO, which was a significant difference compared to younger patients [14]. Similarly, in our study, patients aged 60 years or older showed a significantly lower CR rate for ePRO than those younger than 60 years. However, considering that 87% (334/384) of the older participants completed the questionnaires, the ePRO acceptance using our system appears favorable even among older patients. In our study, ePRO was requested from all patients attending our department, without selection based on their smartphone possession or daily internet use. Furthermore, given that more than 90% of Koreans aged 60 years or older use smartphones [29,30] and most are reported to be familiar with KakaoTalk [31], the ePRO acceptance among our older participants likely reflects the real-world feasibility of implementing ePRO in clinical practice for older Korean patients, particularly when it is delivered through the familiar messaging app. In the meantime, we also found that 13% of patients in this older age group did not properly respond to ePRO, suggesting that there is room for improvement in enhancing ePRO adherence among older patients. It is uncertain why these patients did not respond to the ePRO measurements, as we did not assess the reasons for nonresponse to the questionnaires. However, referencing previous studies, various factors have been identified that affect ePRO acceptance in older patients, including frailty level, socioeconomic status, technological barriers, and the modes of ePRO administration [13,30,32]. Future studies are needed to determine the causes of nonadherence to ePRO and to provide the most appropriate ePRO collection modalities based on the individual characteristics of older Korean patients undergoing cancer treatments.

### Optimizing ePRO Response Through Timing

Interestingly, we found that the timing of ePRO requests was significantly related to the patient’s CR rate. The most appropriate time for requesting ePRO questionnaires was either more than 1 hour before the appointment or in the afternoon on the day before the scheduled visit. This finding suggests that patients may feel more comfortable and have sufficient time to review and respond to ePRO requests when they are delivered within this timeframe. Delivering ePRO questionnaires more than 2 days before a scheduled visit may have hindered appropriate responses, possibly due to difficulties in locating our ePRO request message among other personal messages. As our patients’ ePROs were collected remotely using a mobile app and their completeness was rechecked on-site in our clinic, it is likely that our ePRO acceptance rate is higher than that reported in settings relying solely on remote ePROs collection. This may be indirectly supported by the CR rate of 76.9% (100/130) among our patients who received ePRO questionnaires more than 2 days before an appointment. However, from another perspective, the combination of remote ePRO collection via a messaging app, requested within a specific timeframe, and on-site feedback appears to be an effective strategy for maximizing ePRO acceptance, as indicated by a CR rate of more than 93.8% (547/583) among our patients.

### Strengths and Limitations

A key strength of this study is the provision of real-world data on ePRO adherence and the identification of significant factors influencing adherence among 1488 patients with nonmetastatic breast cancer following implementation of an in-house ePRO platform. Because our ePRO platform uses a messaging app that is largely familiar to Koreans, we observed a favorable acceptance for the ePRO. However, we acknowledge the limitations of this study. Our data were retrospectively derived from a single institution over a 1-year data collection period. Therefore, the data may be insufficient to capture long-term ePRO adherence among general patients with breast cancer. In addition, reasons for PR or NR to ePRO questionnaires were not available, as this analysis was conducted retrospectively. Because the causes of incomplete ePRO are important for identifying areas of improvement in enhancing ePRO adherence, further assessments are necessary among those submitting PR or NR to ePRO questionnaires in future studies. Furthermore, because our ePRO platform relies on a specific mobile messaging app—KakaoTalk, which is predominantly used in South Korea—its general acceptance in other countries remains uncertain. Therefore, the generalizability of our findings to other populations with different digital habits may be limited. In addition, technological barriers or disparities in mobile access may have also influenced patient participation and response accuracy. Moreover, as all our ePRO data were self-reported, reporting bias may have been introduced. Future research involving institutions that use different platforms or serve more diverse populations is needed to explore the generalizability of our findings.

### Conclusions

The collection of ePRO data and its integration into EHR was successful with our ePRO platform, achieving an overall CR rate of 89.9% (1338/1488). Patient age and the specific timeframe for ePRO requests were significant factors influencing complete ePRO acceptance. Patients aged 60 years or older showed significantly lower ePRO adherence. In addition, a specific timeframe, including more than 1 hour before a clinical visit or in the afternoon on the day before the appointment, was associated with a significantly higher CR rate for ePRO. These factors are expected to improve ePRO acceptance among patients with nonmetastatic breast cancer.

## Appendix

Multimedia Appendix 1 Screenshots illustrating the workflow of the electronic health record–integrated electronic patient-reported outcome system and the patient questionnaire interface.

## Abbreviations

CR: complete response
EHR: electronic health record
ePRO: electronic patient-reported outcome
NR: no response
PR: partial response
PRO: patient-reported outcome

## Data Availability

The datasets generated or analyzed during this study are available from the corresponding author on reasonable request.
