# Supplementary material for: Evaluating the Feasibility of an Electronic Patient-Reported Outcomes Platform Integrating Electronic Health Records and a Mobile Messaging App in Breast Cancer Radiotherapy: Retrospective Cross-Sectional Study
Source: JMIR Mhealth Uhealth. 2026 Feb 5;14:e67514. doi: 10.2196/67514 (PMC12921431; doi:10.2196/67514)

**Multimedia Appendix 1**

**Figure S1**. Workflow of the electronic patient-reported outcomes system integrated with electronic health records: (a) requesting the ePRO questionnaire, (b) reviewing the ePRO response

(a)

**
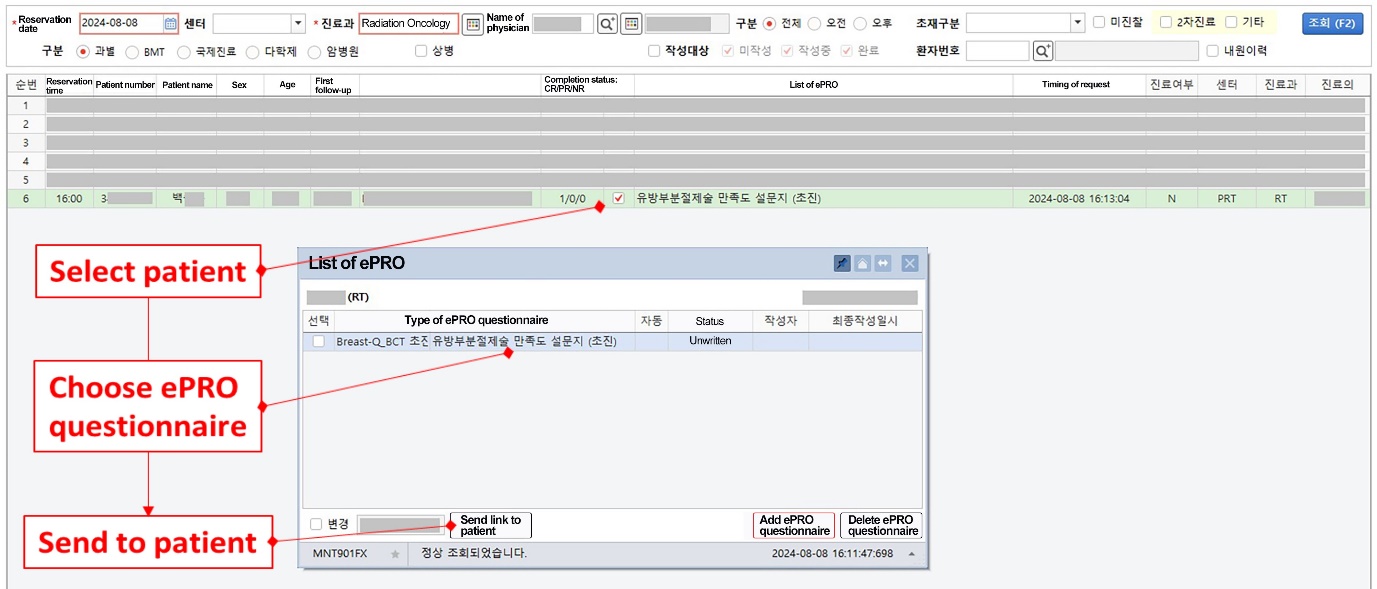
**

(b)


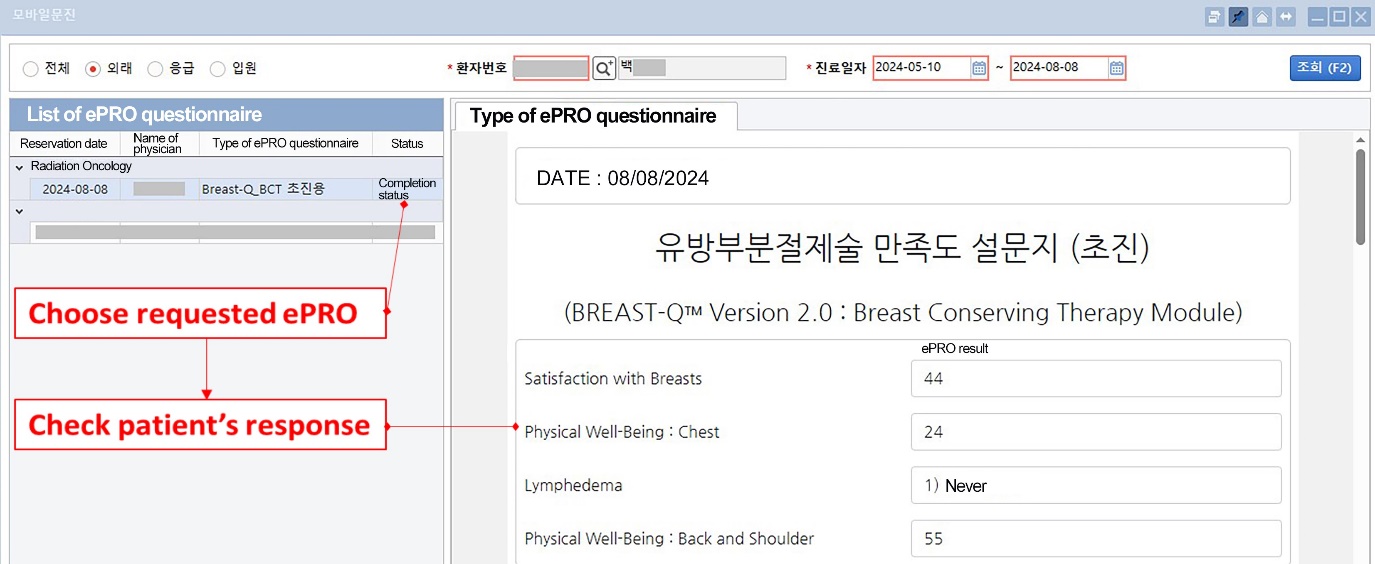


**Figure S2**. Patient interface for completing electronic patient-reported outcomes through the KakaoTalk, mobile messaging application.


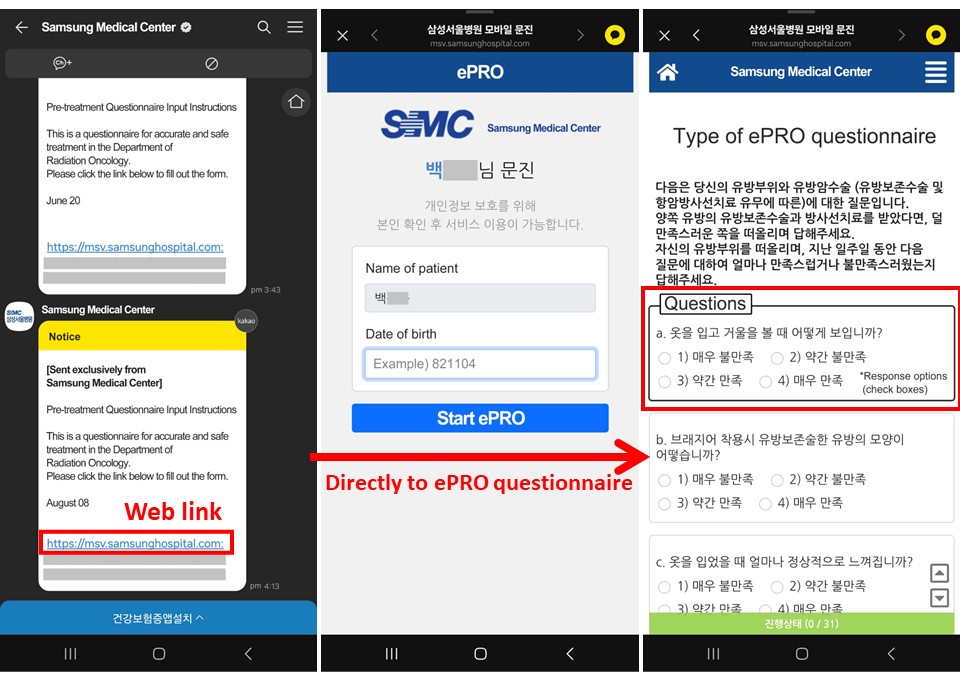

Supplement: Multimedia Appendix 1 [file mhealth_v14i1e67514_app1.docx]
